# Effects of iron nanoparticles and electro-stimulation on biohydrogen production by *Clostridium pasteurianum*

**DOI:** 10.1007/s00253-026-13951-6

**Published:** 2026-07-15

**Authors:** Danielli Azevedo Monico, Guilherme Bueno Bresciani, Adalgisa Rodrigues de Andrade, Valeria Reginatto

**Affiliations:** Department of Chemistry, Faculty of Philosophy, Sciences and Letters of Ribeirão Preto, University of São Paulo, Avenida Bandeirantes, 3900, Ribeirão Preto, 14040-900 São Paulo State Brazil

**Keywords:** Electro-fermentation, Carbon black, Iron nanoparticles, Hydrogen, Organic acid

## Abstract

**Abstract:**

Biological hydrogen (H₂) production is a promising alternative to fossil-based energy processes, and strategies capable of improving H₂ yields are required to enhance its industrial feasibility. In this study, iron nanoparticles supported on carbon black (Fe/CB) were evaluated, for the first time, alone and in combination with electro-stimulated fermentation (EF), to enhance H₂ production by *Clostridium pasteurianum* DSMZ 525. Suspended Fe/CB and Fe/CB immobilized on the anode were employed and compared with traditional fermentation (TF) and EF. Under TF conditions, suspended and immobilized Fe/CB increased H₂ production by 16.7% and 62.5%, respectively, while increasing organic acid concentrations by 3.6- and 4.7-fold. The highest H₂ concentration (129.82 mmol L⁻^1^) and H₂ yield were achieved under EF (0.4 V) without Fe/CB. Under this condition, organic acid and cell biomass concentrations decreased by 83% and twofold, respectively. In contrast, the addition of suspended or immobilized Fe/CB during EF decreased H₂ production by 29% and 24%, respectively, while substantially increasing organic acid concentrations. Overall, Fe/CB enhanced H₂ production during TF, particularly when immobilized on the anode, whereas EF alone proved to be the most effective strategy for maximizing H₂ production. These findings demonstrate that electro-stimulation can redirect microbial metabolism toward H₂ evolution, while Fe/CB supplementation may be more suitable for promoting the formation of value-added fermentation products. The results provide new insights into the combined use of conductive materials and electro-fermentation for controlling product selectivity in *Clostridium*-based bioprocesses.

**Key points:**

- *Suspended iron nanoparticles increase hydrogen production by 16.7% in fermentation.*
- *While immobilized iron nanoparticles increase hydrogen production by 62.5%.*
- *Electro-stimulation yields more hydrogen than iron-assisted electro-stimulation.*

**Supplementary Information:**

The online version contains supplementary material available at https://doi.org/10.1007/s00253-026-13951-6.

## Introduction

Molecular hydrogen (H_2_) plays a pivotal role in advancing both current and future transportation systems. Efforts to expand the use of H_2_-powered vehicles and to establish H_2_ refueling infrastructure are increasing (Trchounian et al. 2017). H_2_ is regarded as the most promising and important energy carrier for the future: it can reduce environmental pollution because its combustion yields only water and energy (Chaubey et al. 2013; Chen et al. 2021; Wang and Yin 2021), and it possesses the highest energy content per unit mass (120 kJ g^−1^) when compared to fossil fuels such as petroleum (42 kJ g^−1^) and coal (24 kJ g^−1^) (Constante Fonseca et al. 2019). Over 90% of commercial H_2_ is produced via methane steam reforming, coal gasification, or water electrolysis. In other words, H_2_ production currently relies on thermochemical processes that directly or indirectly depend on fossil fuels and large amounts of energy, which renders them unsustainable (Chaubey et al. 2013; Chen et al. 2021).

In this context, biological H_2_ production has been considered a promising, low-cost, renewable, and environmentally friendly alternative (Chen et al. 2021). Biological processes require less energy given that they are conducted under ambient temperature and pressure. Moreover, the fermentation route can generate H_2_ from renewable carbohydrate-rich feedstocks including wastewater, lignocellulosic biomass, and algal biomass (Giraldeli et al. 2019).

Fermentative H₂ production from carbohydrates by acidogenic Clostridium species depends on a complex network of iron-containing proteins involved in intracellular electron transfer. Iron is an essential component of pyruvate oxidoreductase (PFOR), [FeFe]-hydrogenase, [NiFe]-hydrogenase, and the electron carrier ferredoxin, all of which play key roles in hydrogen metabolism. Hydrogenases are metalloproteins that catalyze the reversible interconversion between molecular hydrogen and protons and electrons. Among them, [FeFe]-hydrogenases are primarily responsible for proton reduction and H₂ evolution, whereas [NiFe]-hydrogenases are generally associated with hydrogen uptake and oxidation (Hallenbeck 2009; Mulder et al. 2011; Lacasse and Zamble 2016). During dark fermentation, PFOR catalyzes the oxidation of pyruvate to acetyl-CoA and CO₂ while simultaneously reducing ferredoxin. Reduced ferredoxin subsequently transfers electrons to [FeFe]-hydrogenase, which catalyzes the reduction of protons (2H⁺) to molecular hydrogen (H₂) (Angenent et al. 2004; Wang and Yin 2021; Mostafa et al. 2022). In addition, NADH oxidoreductase (NFOR) can regenerate reduced ferredoxin using NADH generated during glycolysis, providing additional reducing equivalents for hydrogen production. Therefore, ferredoxin and [FeFe]-hydrogenase act as central components of the electron transfer network that maintains intracellular redox balance and drives biohydrogen formation. Because iron is a structural and catalytic constituent of these key enzymes and electron carriers, its availability directly influences electron flux toward hydrogen production. Consequently, iron supplementation has been widely investigated as a strategy to enhance H₂ production by *Clostridium* species (Wang and Yin 2021).

Iron nanoparticles (FeNPs) have been extensively investigated as additives to enhance fermentative H₂ production and to modulate microbial metabolism (Lin et al. 2016; Yang and Wang 2018; Moura et al. 2020). Different iron-based nanomaterials, including zero-valent iron, magnetite (Fe₃O₄), hematite, and metallic iron nanoparticles, have been reported to increase hydrogen production by Clostridium species and mixed microbial cultures. Several mechanisms have been proposed to explain these effects, including the release of bioavailable iron for the synthesis and activation of iron-dependent enzymes, reduction of the oxidation–reduction potential of the culture medium, stimulation of ferredoxin-mediated electron transfer, and enhancement of hydrogenase activity (Lin et al. 2016; Zhang et al. 2019; Mishra and Pradhan 2024). For example, Moura et al. (2020) reported that the addition of metallic iron nanoparticles increased H₂ production by approximately 28% during glucose fermentation by *Clostridium butyricum*. Likewise, Zhang et al. (2019) observed enhanced hydrogen production by *Clostridium pasteurianum* in the presence of Fe₃O₄ nanoparticles. Despite these promising results, most studies have focused on nanoparticles dispersed in suspension, whereas supported and immobilized iron nanomaterials remain poorly explored. The exact mechanisms through which these NPs interact with microorganisms remain uncertain. Some possible advantages of using NPs instead of salts include facilitated electron transfer between enzymes. This promotes not only H₂ production, but also synthesis of other fermentation products given that NFOR, PFOR, [FeFe]-hydrogenase, and ferredoxin are in the cytosol (Yang and Wang 2018; Moura et al. 2020; Mishra and Pradhan 2024; Patel et al. 2018; Meinecke et al. 1989).

Conductive carbon materials have also attracted increasing attention in fermentation and bioelectrochemical systems because they can facilitate electron transfer and provide attachment sites for microbial cells. Materials such as activated carbon, biochar, carbon cloth, carbon felt, carbon nanotubes, and carbon black have been reported to influence microbial metabolism by promoting extracellular electron transfer and modifying the local redox environment (Logan et al. 2019; Kumar et al. 2018; Virdis et al. 2022). In addition to their conductive properties, carbon-based supports provide large surface areas for nanoparticle dispersion, potentially improving catalyst stability and accessibility. Therefore, supporting iron nanoparticles on conductive carbon matrices may combine the catalytic role of iron with the electron-conducting properties of carbon materials, creating favorable conditions for hydrogen-producing microorganisms.

Because electro-fermentation and iron supplementation both affect microbial redox metabolism, combining conductive carbon-supported iron nanoparticles with electrochemical stimulation may represent a promising strategy to further improve H₂ production. However, the interaction between these approaches remains largely unknown. Furthermore, the spatial configuration of iron nanoparticles may strongly influence their interaction with microbial cells. Suspended nanoparticles are freely dispersed throughout the culture medium and may increase the probability of direct cell–particle contact and iron uptake. In contrast, immobilized nanoparticles remain confined to the electrode surface, where they may promote biofilm formation, prolong microorganism–material interactions, and facilitate localized electron transfer processes. Previous studies have shown that *C. pasteurianum* can interact with conductive electrodes and form biofilms under electrochemical conditions (Choi et al. 2014; Mostafazadeh et al. 2016), suggesting that immobilized nanomaterials may affect microbial metabolism differently from suspended nanoparticles.

Although iron nanoparticles have been widely investigated for dark fermentative H_2_ production, studies have predominantly focused on suspended nanoparticles. To the best of our knowledge, no study has evaluated iron nanoparticles supported on carbon black (Fe/CB) for hydrogen-producing electro-fermentation, nor compared the effects of suspended and electrode-immobilized Fe/CB configurations. Therefore, this study evaluates, for the first time, the individual and combined effects of Fe/CB and electro-stimulated fermentation on H₂ production by *C. pasteurianum* DSM 525. By comparing suspended and immobilized Fe/CB under both traditional fermentation and electro-fermentation conditions, this work provides new insights into how nanomaterial configuration and electrochemical stimulation influence microbial H_2_ production and metabolite formation.

## Materials and methods

### Traditional fermentation and electro-fermentation assays

#### Inoculum and culture medium

*Clostridium pasteurianum* DSM 525 was obtained from the *Deutsche Sammlung von Mikroorganismen und Zellkulturen* culture collection and used to produce H_2_. Inoculum preparation comprised three stages: microorganism activation, pre-inoculum, and the inoculum itself. Microorganism activation consisted of removing the microorganism cells from the freezer at − 80 °C and transferring 1 mL of the *C. pasteurianum* DSM 525 stock culture stored in Reinforced Clostridium Medium (RCM) supplemented with 30% (v/v) glycerol to fresh RCM. To adapt the microorganism to the culture medium that was used during the fermentation assay, 5 mL of the activated culture was transferred to the experimental culture medium, which was incubated at 37 °C and agitated at 150 rpm for 18 h. In the final stage, after the microorganism was growth in the culture medium, a volume that was sufficient to achieve an optical density (OD) of 0.1 at 600 nm, was added to inoculate the fermentation assay. After inoculation, the microorganism culture was maintained at 37 °C and 150 rpm until the end of the assays.

For the fermentation and electro-fermentation assays, MY culture medium with (g L^−1^) glucose, 10; KH₂PO₄, 3; yeast extract, 3; NaCl, 0.5; MgSO₄·7H₂O, 0.49; Na₂HPO₄, 5.07; NH₄Cl, 1; and CaCl₂, 0.011 was used, as described in Cai et al. (2010). After the medium was prepared, the pH was adjusted to 7.2, and nitrogen gas (N₂) was purged to remove dissolved oxygen (O₂) and to ensure anaerobic conditions. After the medium was prepared, the pH was adjusted to 7.2, and nitrogen gas (N₂) was purged to remove dissolved oxygen (O₂) and ensure anaerobic conditions. The medium was then sterilized by autoclaving at 121 °C for 20 min.

#### Fermentation and electro-fermentation assays

Fermentation assays were conducted in 120-mL flasks. Each flask was filled with 80 mL of MY culture medium and purged with N₂ for 5 min to remove dissolved O₂. The flasks were sealed with tailored rubber stoppers. For electro-fermentation, the rubber stoppers contained a carbon cloth anode (3 × 3 cm^2^) and a platinum cathode (80 × 0.5 mm^2^). The flasks were sterilized by sealing them with the stoppers and autoclaving them at 121 °C and 1 atm for 20 min.

Before the assays were initiated, 8 mL of a 125 g L^−1^ glucose solution was added, so that a final Fe/CB concentration of 10 g L^−1^ was reached. A sufficient volume of inoculum was added so that an initial OD₆₀₀ of 0.1 was achieved. The final volume was completed to 100 mL with MY culture medium (previously degassed with N₂ and sterilized); 20-mL headspace was maintained. Fe/CB were introduced into the systems either as a suspension or immobilized on the anode. In the former case, a Fe/CB suspension was prepared in a flask by adding 4 mg of Fe/CB to 15 mL of previously autoclaved MY culture medium. To remove dissolved O₂, N₂ gas was bubbled through the flask for 5 min, and then the flask was sealed and placed in an ultrasonic bath for 10 min. This ensured that Fe/CB was completely dispersed in the culture medium, which provided greater homogeneity. Finally, 4 mL of Fe/CB suspension was added to the fermentation flask, which furnished the final Fe/CB concentration of 10.7 mg L^−1^. This concentration was selected based on preliminary experiments, as it provided the best microbial growth and fermentation performance among the concentrations evaluated. For the control condition, a CB suspension was prepared by following the same procedure that was used to prepare Fe/CB. To this end, 4 mg of CB was added to 15 mL of MY culture medium, and 4 mL of the resulting suspension was added to the fermentation flask. All fermentation and electro-fermentation assays were performed in triplicate.

For the immobilized Fe/CB, a 3 × 3 cm^2^ carbon cloth was coated with Fe/CB ink prepared from the previously synthesized FeNPs. For this purpose, 4 mg of Fe/CB was mixed with 332.5 µL of isopropanol and 17.5 µL of perfluorinated Nafion™ resin solution (conductive polymer; Sigma-Aldrich, USA). The solution was sonicated for 5 min to ensure that it was completely homogenized. Then, 100 µL of the ink was carefully cast dropwise onto one side of the carbon cloth (3 cm × 3 cm) and left to dry for 30 min, to allow the solvent to be completely evaporated. This procedure allowed 0.12 mg of Fe/CB to be immobilized per cm^2^ of electrode.

The flasks were kept in an incubator at 37 °C and 150 rpm. A potential difference (ΔV) of 0.4 V was applied to the electro-fermentation system. A regulated power supply (HIKARI – HF-3205S) was used; the negative terminal was connected to the carbon cloth electrode, and the positive terminal was connected to the platinum electrode.

**Fig. 1:**
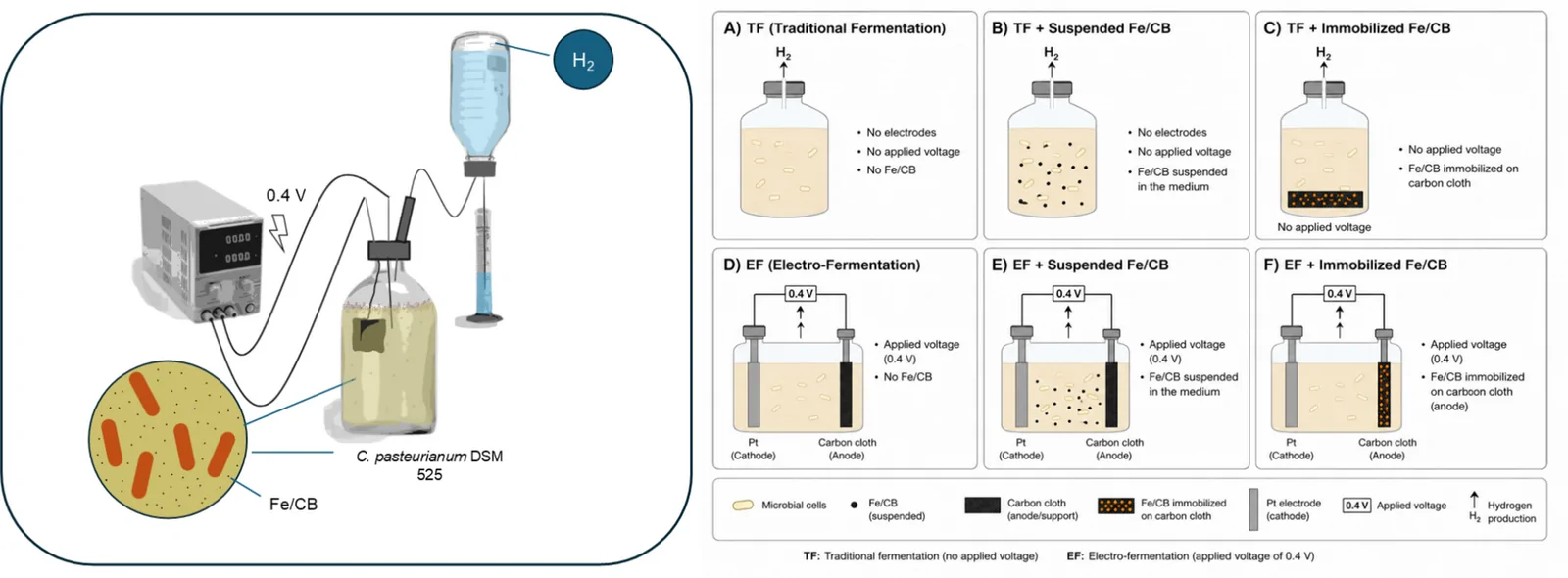
Experimental setup and configurations evaluated for H_2_ production by *Clostridium pasteurianum* DSM 525. The left panel illustrates the electro-fermentation reactor, consisting of a platinum cathode, a carbon cloth anode, an external power supply (0.4 V), and a gas collection system. The right panel summarizes the six experimental conditions investigated: (**A**) traditional fermentation (TF), (**B**) TF with suspended Fe/CB, (**C**) TF with immobilized Fe/CB, (**D**) electro-fermentation (EF), (**E**) EF with suspended Fe/CB, and (**F**) EF with immobilized Fe/CB. Black dots represent Fe/CB dispersed in the culture medium, whereas the orange coating indicates Fe/CB immobilized on the carbon cloth support/electrode

Figure 1 presents the electro-fermentation reactor and summarizes all experimental configurations evaluated in this study, including traditional fermentation (TF), electro-fermentation (EF), and the different Fe/CB application modes (suspended and immobilized).

Control assays without added microorganism were carried out under the following conditions: MY culture medium and application of 0.4 V; MY culture medium containing Fe/CB and application of 0.4 V; and MY culture medium containing CB solution (without FeNPs) and application of 0.4 V. The control assays were also performed under traditional fermentation conditions (without applied potential).

#### Synthesis of iron nanoparticles

FeNPs supported on CB (Fe/CB) were synthesized according to the methodology developed by Bresciani et al. (2023). Initially, 80 mg of Vulcan XC-72R CB thermally pretreated at 400 °C for 4 h and 45 mg of ferrous chloride (FeCl₂) were dissolved in 50 mL of ethylene glycol in a 250 mL round-bottom flask. Dissolution was aided by vortexing, which was followed by ultrasonic bath treatment for 20 min. After the dissolution, the solution pH was adjusted to 11.0 by using 1 mol L^−1^ NaOH in ethylene glycol. The solution was then transferred to a microwave reactor (FlexiWAVE Advanced Flexible Microwave Synthesis Platform), heated from room temperature (25 °C) to 160 °C over 1 min, and kept at 160 °C for 10 min. After cooling, the resulting suspension was sequentially vacuum filtered with ethylene glycol, water, and ethanol. The material retained by filtration was dried in an oven for 18 h, ground, and weighed. A control CB sample was also prepared by following the same methodology without adding FeCl₂.

#### Characterization of iron nanoparticles

Iron was directly quantified in Fe/CB by using the *o*-phenanthroline method, whereby the iron concentration is proportional to the absorbance measured at 510 nm on a UV–Vis spectrophotometer; the absorbance values were correlated with the iron concentration on the basis of a standard curve. For this assay, 5 mg of Fe/CB was resuspended in 6 mL of nitric acid, diluted to 25 mL with ultrapure water, and left to stand overnight. Then, 2 mL of the resulting suspension was mixed with 1 mL of hydroxylamine hydrochloride reducing solution (5%), 2 mL of *o*-phenanthroline complexing agent (1 mmol L^−1^), and 5 mL of acetate buffer solution (2 mol L^−1^, pH 4.0). Iron standards at concentrations of 0.4, 0.8, 1.6, and 2.4 mg L^−1^ were used, to yield the calibration curve: y = 0.4708x + 0.0719; *R*^2^ = 0.9919.

Thermogravimetric analysis (TGA) was conducted by using 4 mg of Fe/CB on a Thermal Analysis System – model Q600 SDT (TA Instruments, New Castle, Delaware, USA) under synthetic air atmosphere, with a heating ramp of 10 °C min^−1^ up to 800 °C.

Fe/CB samples were analyzed by X-ray diffraction (XRD) on a D2 Phaser X-ray diffractometer (Bruker AXS, Billerica, Massachusetts, USA) operated with a copper X-ray source (λ = 0.15406 nm) at 30 kV and 10 mA. Scanning was carried out from 10° to 100° with a step size of 0.05° s^−1^.

#### Cyclic voltammetry experiments

Cyclic voltammetry was conducted to verify whether the anode was modified with Fe/CB. Experiments were performed between − 0.5 and 1.0 V, at a scan rate of 5 mV s^−1^, by using an electrochemical cell containing an Ag/AgCl (saturated) reference electrode, a carbon cloth working electrode, a platinum counter electrode, and MY culture medium (section Inoculum and Culture Medium) with glucose (10 g L^−1^). Measurements were carried out on an AUTOLAB PGSTAT 30 potentiostat (Metrohm, Riverview, FL, USA) controlled by the NOVA 2.1.6 software.

Subsequently, cyclic voltammetry was performed between − 0.5 and 1.0 V, at a scan rate of 5 mV s^−1^, in the EF chamber containing an uncoated or a coated (Fe/CB) carbon cloth working electrode. The assay was conducted in the absence and presence of *C. pasteurianum* after fermentation for 6 h.

#### Hydrogen production quantification and modelling

The gases generated in the fermentation assays were quantified by using an inverted bottle system, as described by Aquino et al. (2007). In this setup, the gases produced during the fermentation process were directed to an inverted 500-mL Duran bottle containing a 5% (w/v) NaOH solution and the pH indicator bromothymol blue. From this system, one hose was connected to the fermentation assays through a needle, while another hose was immersed in NaOH solution of the same concentration inside a 100-mL graduated plastic cylinder. In this way, the volume displaced by the produced gases caused the NaOH solution to be displaced into the cylinder, which allowed the volume of produced gas to be measured. This system was chosen because carbohydrate metabolism by *C. pasteurianum* can generate only H₂ and CO₂ as gaseous products during fermentation. When CO₂ comes into contact with water and NaOH, it is consumed, to form sodium carbonate. Therefore, the system allows the volume of produced H₂ to be exclusively and directly quantified, which was confirmed by gas chromatography.

The modified Gompertz model (Eq. (1)) was used to estimate the kinetic variables of the assays (Table 1):

1 $$H= {H}_{max} \bullet \mathrm{exp}\left\{-\mathrm{exp}\left[\frac{{R}_{m}\bullet e}{{H}_{max}}\left(\lambda -t\right)+1\right]\right\}$$

where **H** is the cumulative H₂ volume in the assays, **Hmax** is the maximum H₂ production potential (mL), **Rm** is the maximum H₂ production rate (mL h^−1^), **λ** represents the lag phase, and **t** is the duration of the assays.

**Table 1:** Kinetic parameters obtained by using the modified Gompertz model, as well as yield factors, in traditional fermentation and electro-fermentation assays of glucose by *C. pasteurianum*

		P (mL)	H_max_ (mL)	R_m_ (mL/h)	λ	r^2^
Traditional Fermentation	**TF**	173.5 ± 68.5 ^b^	203.74 ± 36 ^a^	18.42	16.06 ± 1.87 ^a^	0.9957
	**Susp. Fe/CB**	202.5 ± 3.5 ^ab^	262.04 ± 9.95 ^b^	14.00	14.17 ± 0.41 ^ab^	0.9991
	**CB**	30 ± 24^d^	30.19 ± 0.80 ^d^	3.95	7.76 ± 0.16 ^abc^	0.9915
	**Immob. Fe/CB **	282 ± 18^e^	286.96 ± 5.08 ^be^	33.43	10.09 ± 0.11 ^abcd^	0.9964
Electrostimulated Fermentation	**EF**	330 ± 34 ^f^	457.01 ± 31.29 ^f^	18.93	13.54 ± 0.83 ^abcde^	0.9963
	**Susp. Fe/CB**	232.5 ± 33.5 ^ac^	237.10 ± 3.58 ^bc^	20.33	6.75 ± 0.11 ^abcdef^	0.9975
	**CB**	145 ± 1^bg^	179.23 ± 28.47 ^ag^	9.73	12.84 ± 1.71 ^abcdefg^	0.9676
	**Immob. Fe/CB **	248 ± 9 ^ch^	250.42 ± 4.13 ^bch^	33.77	9.92 ± 0.1 ^abcdefgh^	0.9966

***Hmax*** maximum H₂ volume, ***Rm*** maximum H₂ production rate, ***λ***, lag phase, *Glu* glucose, Initial glucose concentration: 55.51 mmol·L⁻^1^, *TF* Traditional Fermentation, *EF* Electro-fermentation, *Fe/CB* iron nanoparticle supported on carbon black, *CB* carbon black without nanoparticle, *Susp. Fe/CB* suspended Fe/CB, *Immob. Fe/CB* immobilized Fe nanoparticle, * *letters indicate significant differences between results, as revealed by the Tukey test, p* < 0.05

The gases present in the headspace of the traditional fermentation assays without detectable H_2_ volume, as well as in the traditional and electro-stimulated fermentation assays with detectable H_2_ volume, were analyzed on a gas chromatograph (GC-2014, Shimadzu, Japan) equipped with a thermal conductivity detector (TCD). The column consisted of 5-Å molecular sieves (2.0 m × 4.7 mm), and argon was employed as the carrier gas at a flow rate of 30 mL min^−1^. The injector, column, and detector temperatures were set at 80, 50, and 100 °C, respectively. After fermentation for 6 h, 100 µL of headspace gas was collected by using a gastight syringe (1710N, 100 µL, Hamilton, USA).

#### Substrate and product quantification

The soluble co-products of glucose fermentation, such as butyric acid, acetic acid, and butanol, were determined by high-performance liquid chromatography (HPLC) on a Shimadzu (Japan) apparatus operating under the following conditions: Aminex SPX-87H column, mobile phase consisting of H₂SO₄ (0.005 mol L^−1^), flow rate of 0.6 mL min^−1^ (84 kgf cm^−2^), RID-10 detector (refractive index) for butanol, and a diode array detector operating at 190 and 213 nm for the other metabolites. Samples were collected after 24 h of fermentation and centrifuged at 9000 rpm for 4 min to remove microbial cells. The supernatant was then filtered through a 0.22 μm syringe filter. After filtration, the samples were vortexed, and 170 μL was transferred to an insert placed inside an HPLC vial for chromatographic analysis. Data were acquired and processed by using the Class Vp 6.1 software (Shimadzu, Japan). Quantification was carried out by using standard curves of glucose (0.75 to 20 g L^−1^) and acetic acid, butyric acid, and butanol (0.375 to 10 g L^−1^). Liquid samples were collected immediately after inoculation (0 h) and after 24 h of fermentation for HPLC analysis.

#### Statistical analysis

All experiments were performed in triplicate, and the results are presented as mean ± standard deviation. Statistical analyses were performed using one-way analysis of variance (ANOVA), followed by Tukey’s post hoc test for multiple comparisons among treatments. Differences were considered statistically significant at *p* < 0.05.

## Results

**Fig. 2:**
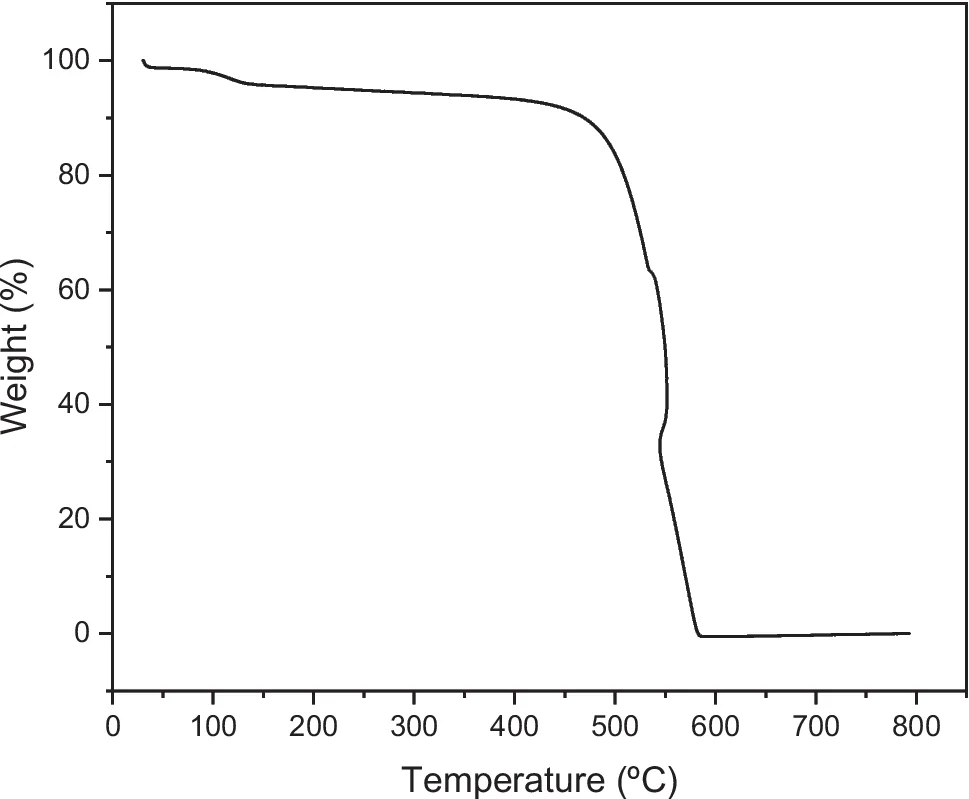
TGA thermograms of Fe/CB samples

Figure 2 shows the TGA thermogram recorded for Fe/CB. The initial mass loss of 3.0% between 50 and 200 °C was characteristic of water and organic solvents, such as ethanol (boiling point: 78.4 °C) and ethylene glycol (boiling point: 197.3 °C), which we used during Fe/CB synthesis and washing (Fellenberg et al. 2021; Liu et al. 2016). The remaining sample underwent mass loss of 93.8% between 400 and 600 °C, which was due to carbon compounds (Fellenberg et al. 2021; Heidarian and Hassan 2021; Losic et al. 2021) and release of CO₂, CO, and CH₄ molecules (Fellenberg et al. 2021). The FeNP mass remaining at the end of the analysis corresponded to 6.2% of the initial mass.

We also quantified the iron content by UV–Vis analysis. The average iron concentration in a 200 mg L^−1^ Fe/CB solution was 11.77 mg L^−1^, which corresponded to 5.9% of FeNPs. This agreed with the TGA-obtained data (6.2%).

**Fig. 3:**
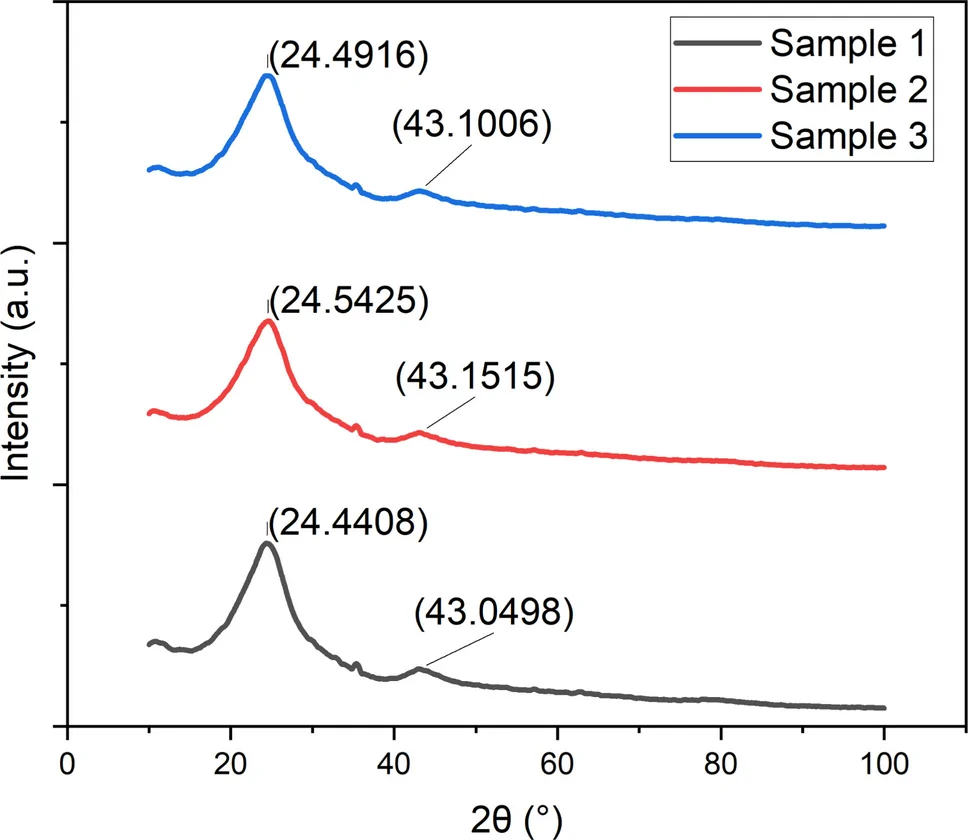
X-ray diffraction (XRD) diffractograms of three Fe/CB samples

Figure 3 presents the diffractograms obtained for different Fe/CB samples. The peak at 20 = 24.4° corresponded to the 0.02 crystallographic plane of carbon (Almeida et al. 2017). The second peak with maximum near 43.1° (2θ) was not well-defined.

### Cyclic voltammetry of nanoparticle-coated electrodes

**Fig. 4:**
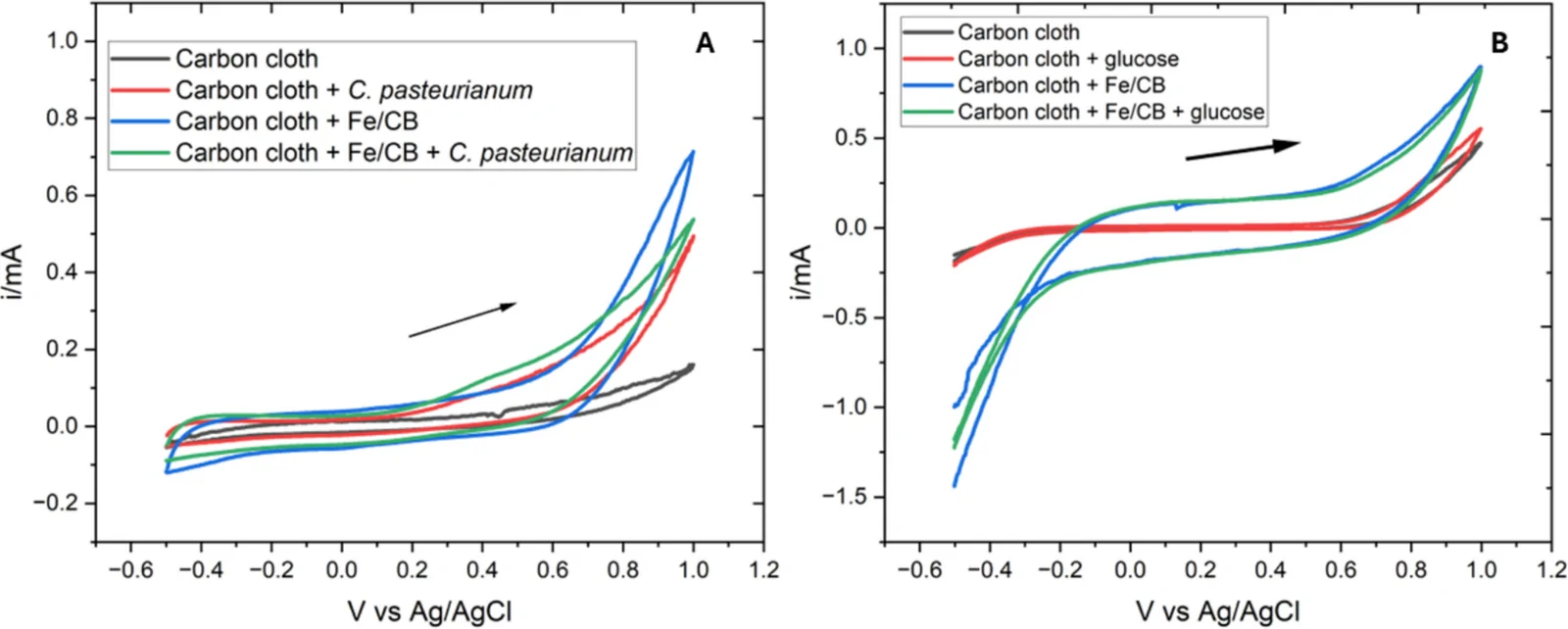
Cyclic voltammetry of Fe/CB immobilized on carbon cloth. (A) Measurements performed under abiotic conditions and in the presence of *C. pasteurianum* in complete culture medium supplemented with 10 g L⁻^1^ glucose. (B) Measurements performed under abiotic conditions in complete culture medium in the presence and absence of 10 g L⁻^1^ glucose. All experiments were conducted at pH 7.2 and a scan rate (ν) of 5 mV s⁻^1^

 We used cyclic voltammetry to monitor how the substrate (glucose), FeNPs, and *C. pasteurianum* behave. First, we conducted the experiment in the presence or absence of glucose for the carbon cloth anode modified with Fe/CB or not. The cyclic voltammograms showed that glucose was not electroactive at any of the anodes Fig. 4B. Figure 4A shows evaluation of the anode modified with Fe/CB under abiotic conditions or in the presence of *C. pasteurianum*.

Under abiotic conditions, the carbon cloth electrodes were not electrochemically active (Fig. 4). When the anodes were modified with Fe/CB, a large pseudo-capacitive region emerged between − 0.4 and + 0.6 V *vs* Ag/AgCl. This is normally associated with increased area, which should allow oxides to be introduced into the electrode (Alexandreli et al. 2021). In addition, we detected potentials more positive than 0.6 V *vs* Ag/AgCl, which corresponds to the onset potential of iron oxidation (Almeida et al. 2017).

The cyclic voltammograms recorded in the presence of *Clostridium* presented a small peak at 0.2 V *vs* Ag/AgCl. Besides that, the current shifted toward less positive potentials.

### Fermentation assays

**Fig. 5:**
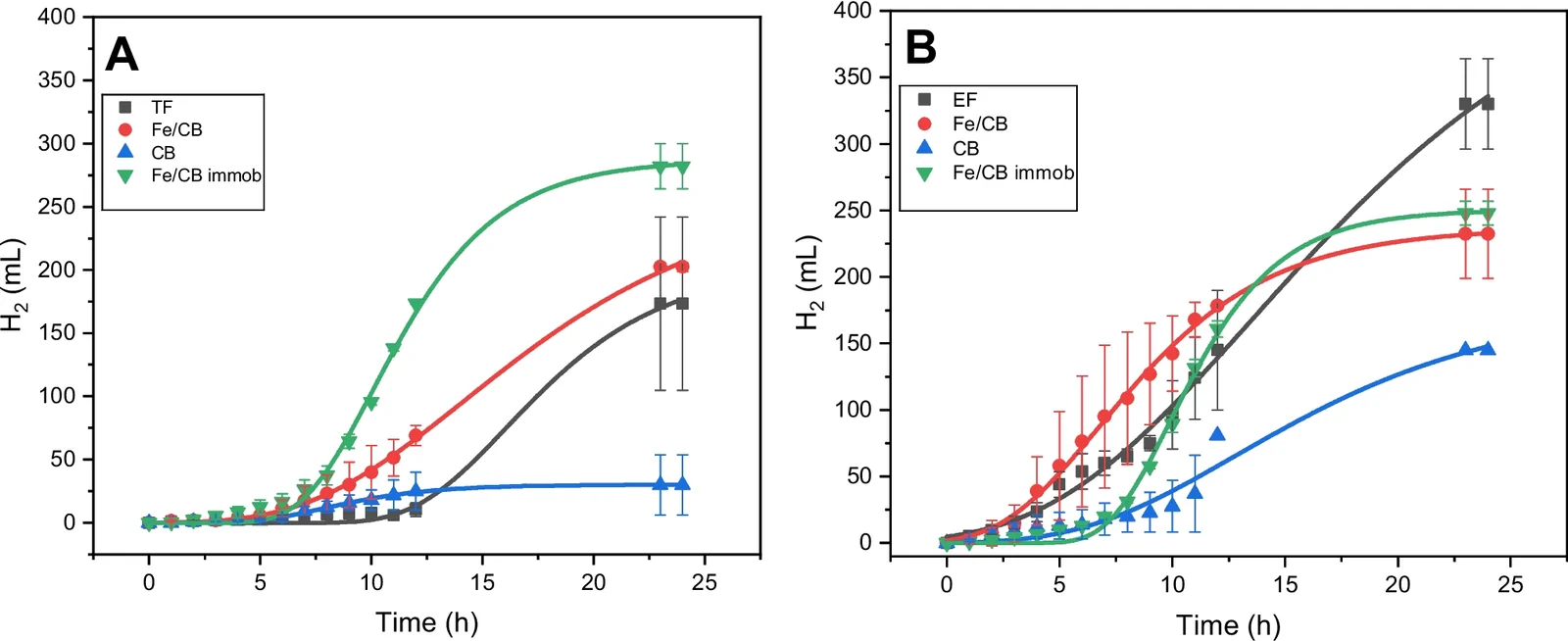
H_2_ volumes (mL) as a function of time obtained during (A) traditional fermentation of glucose by *C. pasteurianum*, and (B) electro-stimulated fermentation of glucose in the same conditions, at applied potential of 0.4 V. Simulation obtained by using the Gompertz model—straight line

Figure 5A shows the H_2_ yields obtained during Traditional Fermentation (TF) of glucose by *C. pasteurianum* in the presence of suspended Fe/CB, CB, or Fe/CB immobilized on the anode. The kinetic parameters estimated by the modified Gompertz model for H_2_ production are presented in Table 1. Table 2 summarizes glucose consumption, absolute pH variation (|ΔpH|), optical density (OD), concentrations of fermentation products, yields, and productivity parameters obtained under the different experimental conditions. We performed control assays to ensure that the quantified gas resulted solely from microorganism metabolism and not from water electrolysis or other elements present in the culture medium. The following conditions—culture medium, glucose solution, and applied potential of 0.4 V; culture medium, glucose solution containing Fe/CB, and applied potential of 0.4 V; and culture medium, glucose solution containing CB, and applied potential of 0.4 V—did not produce any gas. To confirm that H_2_ was produced during TF, we analyzed the gas present in the headspace of the TF assays by gas chromatography (Supplementary Material, Fig. S2). Figure 4B shows H_2_ production during the EF assays.

**Table 2:** Variation in the optical density (OD) and absolute pH (|ΔpH|), glucose consumption, production of soluble glucose fermentation products by *C. pasteurianum*, yields, and productivities after traditional and electro-stimulated fermentation assays conducted for 24 h

		H_2_ (mmol L^−1^)	OD	pH	Glucose consumed (mmol L^−1^)	HAc (mmol L^−1^)	HBu (mmol L^−1^)
Traditional Fermentation	**TF**	68.25 ± 26.95	3.81 ± 0.71	2.1	27.11 ± 5.51^a^	5.64 ± 8.82^c^	5.73 ± 8.06^c^
	**Susp. Fe/CB**	79.66 ± 1.38	6.45 ± 2.90	2.2	52.23 ± 1.11^c^	19.06 ± 1.64^a^	21.31 ± 0.30^b d^
	**CB**	11.80 ± 9.44	1.35 ± 0.92	2.0	19.82 ± 2.67^ab^	6.28 ± 075^cd^	6.42 ± 2.52^c^
	**Immob. Fe/CB**	110.94 ± 7.08	9.05 ± 1.34	2.4	53.95 ± 1.34^c^	26.39 ± 0.27^af^	26.75 ± 0.30^d e^
Electrostimulated Fermentation	**EF**	129.82 ± 13.38	1.79 ± 2.05	0.5	25.31 ± 9.14^abe^	1.94 ± 1.24^b ce^	0.00^a b c^
	**Susp. Fe/CB**	91.46 ± 13.18	3.95 ± 3.04	1.6	25.74 ± 10.68^abde^	11.17 ± 5.53^ab cde^	8.87 ± 9.20^a b c f^
	**CB**	57.04	1.2 ± 1.41	1.9	25.11 ± 10.35^abde^	9.18 ± 3.83^ab cde^	10.37 ± 9.09^b c f^
	**Immob. Fe/CB**	97.56 ± 3.54	10.05 ± 0.35	l2.3	57.63 ± 4.42^a^	25.44 ± 0.05^af^	24.88 ± 0.0^d e^

	BuOH (mmol L^−1^)	Organic acids (mmol L^−1^)	${Y}_{X/Glc}$ (mg mmol^−1^)	${Y}_{{H}_{2}/Glc}$ (mmol mmol^−1^)	${Y}_{HAc/Glc}$ (mmol mmol^−1^)	${Y}_{HBu/Glc}$ (mmol mmol^−1^)	${P}_{{H}_{2}}$ (mmol L^−1^ h^−1^)
Traditional Fermentation	3.18 ± 4.50^a^	11.37 ± 11.95	2.84 ± 0.79	2.52 ± 1.12	0.21 ± 0.37	0.17 ± 0.34	2.84 ± 1.12
	10.48 ± 3.06^ab^	40.37 ± 1.67	2.49 ± 1.125	1.53 ± 0.04	0.36 ± 0.03	0.41 ± 0.01	3.32 ± 0.06
	0.00^abc^	12.70 ± 2.63	1.39 ± 0.97	0.60 ± 0.48	0.32 ± 0.06	0.32 ± 0.15	0.49 ± 0.39
	6.16 ± 0.09^acd^	53.14 ± 0.40	3.39 ± 0.51	2.06 ± 0.14	0.49 ± 0.01	0.50 ± 0.01	4.62 ± 0.30
Electrostimulated Fermentation	0.00^acde^	1.94 ± 1.24	1.44 ± 1.72	5.13 ± 1.93	0.08 ± 0.18	0.00	5.41 ± 0.56
	0.00^abcdef^	20.04 ± 10.73	3.10 ± 2.72	3.55 ± 1.56	0.43 ± 0.39	0.34 ± 0.53	3.81 ± 0.55
	2.81 ± 3.97^abcdefg^	19.55 ± 9.86	0.97 ± 1.21	2.27 ± 0.94	0.37 ± 0.32	0.41 ± 0.53	2.38
	6.18 ± 0.0^abcdefg^	50.32 ± 0.05	3.52 ± 0.3	1.69 ± 0.14	0.44 ± 0.01	0.43 ± 0.01	4.07 ± 0.15

Initial glucose concentration: 55.51 mmol L^−1^

*TF* Traditional Fermentation, *EF* Electro-stimulated fermentation, *Fe/CB* Iron nanoparticles supported on carbon black, *CB *Carbon black, *Susp. Fe/CB* suspended Fe/CB, *Immob. Fe/CB* Fe/CB immobilized on the anode, *HAc* acetic acid, *HBu* butyric acid *BuOH* butanol *Y* yield *P* productivity, *Glc* glucose,

* letters indicate significant differences between the results, as revealed by Tukey’s test, *p* < 0.05

Table 2 lists the products obtained from glucose EF by *C. pasteurianum* after 24 h.

Figure 5A and Table 2 show that Fe/CB positively affected traditional fermentation (TF). H_2_ production increased from 68.25 ± 26.95 mmol L⁻^1^ in the control assay to 79.66 ± 1.38 mmol L⁻^1^ in the presence of suspended Fe/CB and to 110.94 ± 7.08 mmol L⁻^1^ when Fe/CB was immobilized on the anode, corresponding to increases of 16.7% and 62.5%, respectively. In contrast, carbon black (CB) alone reduced H_2_ production to 11.80 ± 9.44 mmol L⁻^1^. The increase in H₂ production observed with Fe/CB was accompanied by higher glucose consumption, which increased from 27.11 ± 5.51 mmol L⁻^1^ in the control to approximately 52–54 mmol L⁻^1^ in the Fe/CB-containing assays. Likewise, total organic acid concentration increased from 11.37 ± 11.95 mmol L⁻^1^ in TF to 40.37 ± 1.67 and 53.14 ± 0.40 mmol L⁻^1^ in the suspended and immobilized Fe/CB systems, respectively.

Electro-fermentation (EF) produced the highest H_2_ concentration among all evaluated conditions. Application of 0.4 V increased H_2_ production to 129.82 ± 13.38 mmol L⁻^1^, representing a 90.2% increase relative to TF. Under these conditions, glucose consumption remained similar to that observed in TF (25.31 ± 9.14 mmol L⁻^1^), whereas organic acid production decreased substantially, from 11.37 ± 11.95 mmol L⁻^1^ in TF to 1.94 ± 1.24 mmol L⁻^1^ in EF. Biomass formation was also lower, with OD decreasing from 3.81 ± 0.71 to 1.79 ± 2.05.

The addition of Fe/CB during EF reduced H_2_ production compared with EF alone. Suspended Fe/CB and immobilized Fe/CB resulted in H₂ concentrations of 91.46 ± 13.18 and 97.56 ± 3.54 mmol L⁻^1^, respectively. However, both configurations increased glucose consumption, biomass formation, and metabolite production. In particular, immobilized Fe/CB increased organic acid concentration from 1.94 ± 1.24 mmol L⁻^1^ in EF to 50.32 ± 0.05 mmol L⁻^1^. Similar trends were observed for acetic and butyric acids, whose concentrations increased from 1.94 and 0.00 mmol L⁻^1^ in EF to 25.44 and 24.88 mmol L⁻^1^, respectively, when Fe/CB was immobilized on the anode.

## Discussion

### Nanoparticles characterization

The agreement between TGA (6.2%) and UV–Vis (5.9%) results confirmed the FeNP content in the Fe/CB material (Fig. 2) (Fellenberg et al. 2021; Heidarian and Hassan 2021; Losic et al. 2021).

The diffractograms obtained (Fig. 3) showed that the peak at 43.1° (2θ) was broad and not well-defined, and the expected peaks at 63.20° and 79.85° (JCPDS-01–071–4650), characteristic of the cubic iron structure (space group Im-3m), were not clearly observed. The broad peak at 43.1° (2θ) may be attributed to poorly crystalline iron-containing phases, including iron(II) oxide (JCPDS-01–091–1715), γ-Fe₂O₃ maghemite (JCPDS-01–089–5192), or iron(III) oxide (JCPDS-01–079–0419) (Fleet 1986; Almeida et al. 2017). However, due to the low intensity and poor definition of this reflection, as well as the absence of other characteristic diffraction peaks, an unambiguous phase assignment was not possible. In addition, the very small size of the FeNPs may have contributed to peak broadening and reduced diffraction intensity. Previous studies using a similar synthetic approach reported Fe nanoparticles with diameters in the range of 1–5 nm, for which diffraction peaks are often weak and broadened (Bresciani et al. 2023). The relatively low iron loading in the composite (approximately 6 wt%), combined with the predominance of carbon black in the sample, may have further hindered the detection of iron-related reflections. Therefore, the weak diffraction features may result from the combined effects of low crystallinity, ultrasmall particle size, and low iron concentration in the composite material (Fiévet et al. 2018).

Overall, the characterization results confirmed the successful incorporation of iron nanoparticles into the carbon black matrix. The close agreement between the iron contents determined by TGA (6.2%) and UV–Vis analysis (5.9%) demonstrated the reliability of the synthesis procedure and the reproducibility of the Fe loading. Furthermore, the broad and poorly defined diffraction peaks observed in the XRD patterns indicated that the FeNPs were predominantly amorphous and highly dispersed on the carbon support. Such structural characteristics are expected to provide a high surface area and a large number of accessible reactive sites, which may favor interactions between FeNPs, microorganisms, and electrode surfaces in the subsequent fermentation and electro-fermentation experiments.

### Cyclic voltammetry of nanoparticle-coated electrodes

The cyclic voltammograms (Fig. 4) showed that, under abiotic conditions, the carbon cloth electrodes were not electrochemically active. When the anodes were modified with Fe/CB, a large pseudo-capacitive region emerged between − 0.4 and + 0.6 V vs Ag/AgCl, which is normally associated with increased area and allows oxides to be introduced into the electrode (Alexandreli et al. 2021). In addition, potentials more positive than 0.6 V vs Ag/AgCl were detected, which correspond to the onset potential of iron oxidation (Almeida et al. 2017).

The cyclic voltammograms recorded in the presence of *C. pasteurianum* (Fig. 4A) presented a small peak at 0.2 V vs Ag/AgCl. Besides that, the current shifted toward less positive potentials, which indicated that an oxidative process took place (Aoki et al. 2020). This behavior became more pronounced in the presence of Fe/CB, which indicated that microbial electroactivity and FeNPs acted synergistically.

Choi et al. (2014) demonstrated the electroactivity of *C. pasteurianum* through direct electron uptake from the electrode and suggested that microbe–electrode interactions can modulate extracellular electron transfer processes. In this context, the electrochemical responses observed herein (Fig. 3) suggested that such interactions modified the local redox environment near the electrode surface, which was amplified by Fe/CB.

### Traditional fermentation assays

Concerning TF, Fig. 4A showed that introducing suspended Fe/CB or Fe/CB immobilized on the anode increased H₂ production. In contrast, introducing CB without FeNPs inhibited H₂ formation by 82.7%, because CB presents cell toxicity, which is associated with its structure and depends on its concentration (Chen et al. 2019; Malode et al. 2024). Carbon-based nanomaterials exert cytotoxicity mainly by disrupting the electrostatic balance of microbial cell membranes or organelles or by generating oxidative stress, which alters lipids, proteins, and cell metabolism, thereby damaging cells (Chen et al. 2019).

It is noteworthy that supporting FeNPs on CB produced positive effects, which could indicate that CB acts as a delivery system, to release FeNPs into the microorganisms. H₂ production decreased as follows: TF with Fe/CB immobilized on the anode > TF with suspended Fe/CB > TF > > TF with CB (Fig. 4A), which showed that the presence of Fe/CB positively affected H₂ production.

Compared to TF and TF with CB, TF with Fe/CB almost doubled the glucose consumption (Table 1). FeNPs (suspended Fe/CB or Fe/CB immobilized on the anode) positively influenced H₂ production and soluble product concentration. Indeed, production of organic acids was almost threefold higher, while the acetic and butyric acid yields were fourfold higher, and the butanol yield was two- or threefold higher when compared to TF.

The enhancement of H₂ production observed in both suspended and immobilized Fe/CB treatments may be associated with the central role of iron in the hydrogen-producing metabolism of *Clostridium pasteurianum*. Iron is a constituent of ferredoxin and [FeFe]-hydrogenase, which are directly involved in electron transfer and proton reduction during dark fermentation. Therefore, Fe/CB may increase iron availability and facilitate electron transfer toward H_2_ evolution. In addition, the conductive carbon black support may promote electron transport, while the immobilized configuration can favor cell-material interactions at the electrode surface. These combined effects may explain why both suspended and immobilized Fe/CB enhanced H₂ production compared with traditional fermentation without Fe/CB.

Compared with conventional iron supplementation using soluble iron salts or iron-containing minerals, Fe/CB may offer advantages due to the high surface area of iron nanoparticles and their potential involvement in electron transfer processes. Moreover, supporting FeNPs on carbon black enables their immobilization on electrode surfaces, which cannot be readily achieved with dissolved iron salts. This feature may be particularly advantageous in electro-fermentation systems, where prolonged interactions among microorganisms, conductive materials, and iron-containing particles can be established. Nevertheless, the results obtained with CB alone indicate that the carbon support may exert inhibitory effects on microbial growth and H₂ production. Therefore, the overall performance of Fe/CB likely reflects a balance between the beneficial effects of FeNPs and the adverse effects associated with the carbon support.

Zhang et al. (2019) observed that H₂ production by *C. pasteurianum* increased by 33% when 100 mg L⁻^1^ magnetite (Fe₃O₄) NPs was added during glucose fermentation. Our results showed that the H₂ volume increased by 16.7% when a much lower content of Fe/CB was added, and that the H₂ volume increased by 62.5% when Fe/CB were immobilized on the anode. This suggested that, although CB may be toxic to cells, supporting FeNPs on CB positively affected TF because iron increases the electrical conductivity and favors electron exchange.

To better contextualize the performance of the present system relative to previously reported iron nanoparticle-assisted and electro-fermentation processes, a comparative summary of representative studies is presented in Table 3.

**Table 3:** Comparison between the present study and previous studies employing iron-based nanoparticles and/or electro-fermentation systems for H_2_ production

Microorganism	Substrate	Process	Nanomaterial/Electrode modification	H_2_production or yield	Improvement (%)	Reference
*Clostridium butyricum*	Glucose	TF	Metallic Fe and Ni nanoparticles	+ 28% H₂ production	28	Moura et al. (2020)
*Clostridium pasteurianum*	Glucose	TF	Fe₃O₄ nanoparticles (100 mg L⁻^1^)	+ 33% H₂ production	33	Zhang et al. (2019)
*Clostridium pasteurianum*	Glucose	TF	Suspended Fe/CB	79.66 mmol L^−1^ H₂	+ 16.7	This study
*Clostridium pasteurianum*	Glucose	TF	Immobilized Fe/CB	110.94 mmol L^−1^ H₂	+ 62.5	This study
*Clostridium pasteurianum*	Glucose	EF (0.4 V)	No FeNPs	129.82 mmol L^−1^ H₂	+ 90.2 vs TF	This study
*Clostridium pasteurianum*	Glucose	EF (0.4 V)	Suspended Fe/CB	91.46 mmol L^−1^ H₂	− 29 vs EF	This study
*Clostridium pasteurianum*	Glucose	EF (0.4 V)	Immobilized Fe/CB	97.56 mmol L^−1^ H₂	− 24 vs EF	This study
*Enterobacter aerogenes*	glucose	TF	Ferric oxide nanoparticles	192.4 mL g^−1^	17	Lin et al. (2016)
Mixed culture	Various substrates	MEC	FeS-modified biocathode	7.01 mol H_2_ mol^−1^ glucose	+ 20.2 vs TF	Qing et al. (2025)
Mixed culture	Grass biomass	TF	zero-valent iron nanoparticles	64.7 mL g^−1^-dry grass	73.1	Yang and Wang (2018)

*TF* traditional fermentation, *EF* electro-fermentation, *Fe/CB* iron nanoparticles supported on carbon black

Since the Gompertz parameters are estimates derived from the mathematical fitting of the model to the experimental data, the maximum H_2_ production values predicted by the model may exceed those observed experimentally. This occurs because the model estimates the production potential based on the overall trend of the data.

### Electro-stimulated fermentation assays

Regarding EF, Fig. 5B showed that EF alone provided the highest accumulated H₂ volume, while EF in the presence of suspended Fe/CB or Fe/CB immobilized on the anode resulted in lower H₂ production. In other words, the applied voltage affected H₂ production more positively than the presence of Fe/CB.

Additionally, the applied voltage hindered the deleterious effect of CB. Thus, H₂ production decreased as follows: EF > EF with Fe/CB immobilized on the anode > EF with suspended Fe/CB > > EF with CB (Fig. 5B).

Previous studies have reported that *C. pasteurianum* can form biofilms on electrode surfaces (Choi et al. 2014; Mostafazadeh et al. 2016), which may explain the increased H₂ production, soluble product formation, and microbial growth observed when Fe/CB was immobilized on the anode. Biofilm formation can enhance and prolong the interaction between microorganisms and Fe/CB, favoring the availability of iron and facilitating electron transfer processes (Table 2 and Fig. 5). Interestingly, immobilized Fe/CB promoted higher H₂ production than suspended Fe/CB, despite the greater dispersion of the latter in the culture medium. This finding suggests that the positive effect of Fe/CB may depend not only on the frequency of particle-cell encounters but also on the duration and stability of these interactions. Immobilization may provide a favorable surface for biofilm development and sustained contact between cells and Fe/CB, while also reducing nanoparticle aggregation and sedimentation. Together, these factors may contribute to the superior performance of immobilized Fe/CB during the fermentation process.

Although both electro-fermentation and Fe/CB supplementation individually enhanced H₂ production, their combination did not result in a clear additive effect. A possible explanation is that both strategies influence similar metabolic processes related to intracellular electron transfer and H_2_ evolution. Electro-fermentation may increase the availability of reducing equivalents through cathodic electron uptake, whereas Fe/CB may facilitate electron transfer and support the activity of iron-dependent proteins involved in H₂ production. Therefore, once the electron-transfer bottleneck is alleviated by one strategy, the additional benefit provided by the other may become limited.

Furthermore, the capacity of the hydrogen-producing pathway may be constrained by the availability and activity of key enzymes such as ferredoxin and [FeFe]-hydrogenase. Under these conditions, increasing electron availability beyond a certain threshold may not lead to proportional increases in H₂ production. In addition, the inhibitory effects observed for carbon black alone suggest that any positive contribution of Fe/CB could be partially offset by adverse effects associated with the support material. Together, these factors may explain the absence of a strong synergistic effect between electro-fermentation and Fe/CB under the conditions evaluated.

EF in the absence of Fe/CB resulted in low OD, low glucose consumption, and low soluble product concentration (Table 2), but yielded the highest H₂ production (Fig. 4B). Thus, applying only the potential difference increased H₂ production probably because microorganism metabolism was modulated. NAD⁺ reduction to NADH by the applied potential, combined with the oxidative action of the NFOR enzyme, may have increased H₂ production without necessarily increasing glucose consumption.

This explains the higher H₂/glucose yield compared to the stoichiometric value. Cardeña et al. (2024) had previously observed this behavior. These authors reported that applying potentials between − 0.5 and 0.4 V increased H₂ production compared to TF. Other studies also reported enhanced yields under applied voltage (Kumar et al. 2018; Bhagchandanii et al. 2020; Qing et al. 2025), suggesting that the positive effect of electro-fermentation on H_2_ production is not restricted to *C. pasteurianum*. Similar responses have been associated with changes in intracellular redox balance and electron availability, which favor proton reduction and H₂ evolution.

Selembo et al. (2009) also observed yields above theoretical values, while the theoretical maximum is lower (Hallenbeck 2009). Their results indicated that EF influenced the NADH/NAD⁺ balance and affected metabolic pathways. The above-mentioned studies also reported that increasing the intracellular pool of NADH or ATP enhanced H₂ production, provided that NAD⁺ was rapidly regenerated (Jiang et al. 2018; Li et al. 2022; Virdis et al. 2022; Narcizo et al. 2025).

The organic acid profile observed in this study was consistent with that commonly reported for glucose fermentation by *C. pasteurianum*, in which acetate and butyrate are the predominant soluble metabolites. These products are typically associated with hydrogen-producing pathways and reflect the distribution of reducing equivalents during fermentation. However, unlike conventional dark fermentation, electro-fermentation resulted in lower concentrations of organic acids despite the higher H₂ yield obtained. This observation suggests that the enhanced H₂ production was not driven by increased acidogenic activity. Instead, it is likely that part of the reducing power required for H₂ evolution was supplied by electrons derived from the cathode, decreasing the need to channel carbon and electrons toward organic acid formation. Therefore, organic acid accumulation did not appear to limit electro-fermentation performance under the studied conditions and may instead indicate a redistribution of electron flux toward H_2_ production.

Choi et al. (2014) also observed decreased OD, glucose consumption, and organic acid concentrations under electrostimulation, which is consistent with the results shown in Table 1. These authors also demonstrated that electron uptake from the cathode induced NADH generation.

Mostafazadeh et al. (2016) also found that the specific growth rate decreased during EF as compared to TF, reinforcing that the applied potential alters microbial metabolism.

**Fig. 6:**
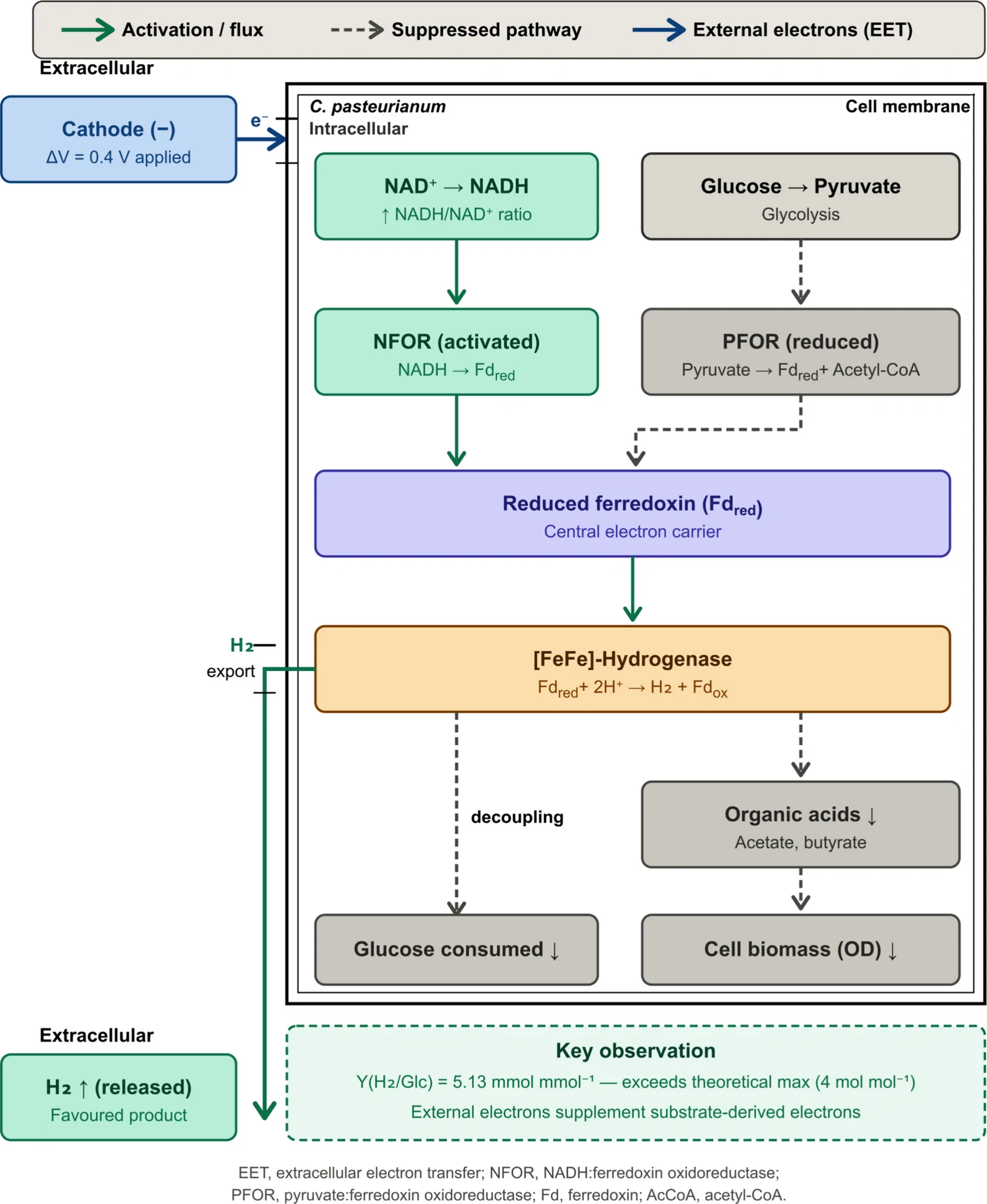
Hypothetical mechanism proposed to explain the enhancement of H₂ production by *Clostridium pasteurianum* under electro-fermentation (0.4 V). Figure generated using artificial intelligence (Claude)0.9

The combination of increased H₂ yield and reduced glucose consumption, biomass formation, and organic acid production suggests that electro-fermentation altered the intracellular electron flow of *C. pasteurianum*. Based on these observations and on previous evidence of cathodic electron uptake by this microorganism (Choi et al. 2014), a hypothetical metabolic model is proposed (Fig. 6). Cathodic electron uptake may increase the intracellular NADH/NAD⁺ ratio and stimulate the formation of reduced ferredoxin through the NFOR pathway. Reduced ferredoxin can subsequently serve as an electron donor for [FeFe]-hydrogenase, promoting proton reduction and H₂ evolution. The proposed mechanism is consistent with the enhanced H₂ production observed under electro-fermentation, despite lower glucose consumption, biomass accumulation, and organic acid formation. These results suggest that external electrons partially supplement the reducing power normally generated from substrate oxidation, thereby increasing electron availability for H_2_ production while reducing carbon flux toward cell growth and fermentation by-products.

Fe/CB immobilized on the anode increased biomass and substrate consumption (Table 1). Therefore, Fe/CB increased H₂ production by stimulating microbial growth. This happened probably because the FeNPs immobilized on the anode surface improved the electrical conductivity (Alexandreli et al. 2021), facilitated electron transfer to the catalysts (Arduini et al. 2020), and enhanced the cathode performance (Sun et al. 2024). Consequently, Fe/CB immobilization improved electron transfer and, together with biofilm formation, increased the contact between microorganisms and FeNPs, thereby improving the overall process efficiency.

Finally, electrostimulation affected H₂ production more positively than the presence of Fe/CB. Coenzyme regeneration under an applied potential of 0.4 V may have modulated microbial metabolism toward the H₂-producing pathway without increasing biomass or organic acid production.

The results obtained in this study highlight important differences between traditional fermentation (TF) and electro-fermentation (EF). Under the evaluated conditions, EF resulted in the highest H₂ production and H₂ yield, while requiring lower glucose consumption and generating lower concentrations of biomass and soluble metabolites. These findings suggest that EF is particularly attractive when maximizing H_2_ production is the primary objective. However, EF requires the application of an external voltage and the use of electrochemical equipment, which may increase operational complexity and capital costs compared with conventional fermentation.

In contrast, TF is a simpler and potentially more scalable process due to its lower operational requirements. Moreover, when coupled with Fe/CB, TF promoted higher microbial growth and organic acid production, indicating that this strategy may be advantageous when organic acids are desired as target products. Therefore, the choice between TF and EF should consider the intended application. While EF appears more suitable for maximizing H₂ production through metabolic modulation and electron supplementation, TF coupled with Fe/CB may represent an attractive alternative for the production of value-added fermentation products.

From an industrial perspective, further studies are required to evaluate the economic feasibility, long-term stability, and scalability of electro-fermentation systems. In particular, the integration of conductive materials and electrochemical stimulation may provide new opportunities for controlling product selectivity and improving process performance in biohydrogen production.

## Conclusion

We have confirmed that electro-stimulating fermentation affects H₂ production positively as compared to traditional fermentation. We achieved the highest H₂ concentration (129.82 ± 13.38 mmol L^−1^) by conducting electro-fermentation in the absence of iron nanoparticles in the system, despite the lower cell biomass (OD of 1.79) compared to other conditions (OD of 3.81 during traditional fermentation). This suggested that microorganism metabolism was modulated toward H₂ production rather than biomass production and formation of other fermentative products. However, immobilizing iron nanoparticles supported on carbon black on the anode during traditional fermentation increased H₂ production (110.94 ± 7.08 mmol L^−1^), microbial growth, and formation of other important fermentation products, including organic acids such as acetic and butyric acids. Carbon black without iron nanoparticles reduced microbial growth and H₂ production, which demonstrated its inhibitory effect on the microorganism.

## Supplementary Information

Below is the link to the electronic supplementary material.

ESM 1 (DOCX 179 KB)

## Data Availability

No datasets were generated or analysed during the current study.

## References

[CR1] Alexandreli M, Brocchi CB, Soares DM, Nunes WG, Freitas BG, de Oliveira FER, Shiavo LECA, Peterlevitz AC, da Silva LM, Zanin H (2021) Pseudocapacitive behaviour of iron oxides supported on carbon nanofibers as a composite electrode material for aqueous-based supercapacitors. J Energy Storage 42:103052. 10.1016/j.est.2021.103052

[CR2] Almeida TS, Garbim C, Silva RG, de Andrade AR (2017) Addition of iron oxide to Pt-based catalyst to enhance the catalytic activity of ethanol electrooxidation. J Electroanal Chem 796:49–56. 10.1016/j.jelechem.2017.04.039

[CR3] Angenent LT, Karim K, Al-Dahhan MH, Wrenn BA, Domínguez-Espinosa R (2004) Production of bioenergy and biochemicals from industrial and agricultural wastewater. Trends Biotechnol 22:477–485. 10.1016/j.tibtech.2004.07.001 15331229

[CR4] Aoki KJ, Chen J, Liu Y, Jia B (2020) Peak potential shift of fast cyclic voltammograms owing to capacitance of redox reactions. J Electroanal Chem 856:113609. 10.1016/j.jelechem.2019.113609

[CR5] Aquino SF, Chernicharo CAL, Foresti E, Santos MLF, Monteggia LO (2007) Metodologias para determinação da atividade metanogênica específica (AME) em lodos anaeróbios. Eng Sanit Ambient 12:192–201. 10.1590/S1413-41522007000200010

[CR6] Arduini F, Cinti S, Mazzaracchio V, Scognamiglio V, Amine A, Moscone D (2020) Carbon black as an outstanding and affordable nanomaterial for electrochemical (bio)sensor design. Biosens Bioelectron 156:112033. 10.1016/j.bios.2020.112033 32174547

[CR7] Bhagchandanii DD, Babu RP, Sonawane JM, Khanna N, Pandit S, Jadhav DA, Khilari S, Prasad R (2020) A comprehensive understanding of electro-fermentation. Fermentation 6:92. 10.3390/fermentation6030092

[CR8] Bresciani GB, Franco JH, Kokoh KB, Napporn TW, de Andrade AR (2023) Improved glycerol electrooxidation at carbon-supported PdFe bimetallic catalysts. J Electrochem Soc 170:124504. 10.1149/1945-7111/ad1066

[CR9] Cai G, Jin B, Saint C, Monis P (2010) Metabolic flux analysis of hydrogen production network by *Clostridium butyricum* W5: effect of pH and glucose concentrations. Int J Hydrogen Energy 35:6681–6690. 10.1016/j.ijhydene.2010.04.097

[CR10] Cardeña R, Valencia-Ojeda C, Chazaro-Ruiz LF, Razo-Flores E (2024) Regulation of the dark fermentation products by electro-fermentation in reactors without membrane. Int J Hydrogen Energy 49:107–116. 10.1016/j.ijhydene.2023.06.253

[CR11] Chaubey R, Sahu S, James OO, Maity S (2013) A review on development of industrial processes and emerging techniques for production of hydrogen from renewable and sustainable sources. Renew Sustain Energy Rev 23:443–462. 10.1016/j.rser.2013.02.019

[CR12] Chen M, Sun Y, Liang J, Zeng G, Li Z, Tang L, Zhu Y, Jiang D, Song B (2019) Understanding the influence of carbon nanomaterials on microbial communities. Environ Int 126:690–698. 10.1016/j.envint.2019.02.005 30875562

[CR13] Chen L, Zhang K, Wang M, Zhang Z, Feng Y (2021) Enhancement of magnetic field on fermentative hydrogen production by *Clostridium pasteurianum*. Bioresour Technol 341:125764. 10.1016/j.biortech.2021.125764 34438289

[CR14] Choi O, Kim T, Woo HM, Um Y (2014) Electricity-driven metabolic shift through direct electron uptake by electroactive heterotroph *Clostridium pasteurianum*. Sci Rep 4. 10.1038/srep06961 PMC4223642 25376371

[CR15] Constante Fonseca B, Schmidell W, Reginatto V (2019) Impact of glucose concentration on productivity and yield of hydrogen production by the new isolate *Clostridium beijerinckii* Br 21. Can J Chem Eng 97:1092–1099. 10.1002/cjce.23327

[CR16] Fellenberg AK, Luchese CL, Marcilio NR, Tessaro IC (2021) Supported carbon membranes using poly(ether sulfone) precursor. Korean J Chem Eng 38:565–575. 10.1007/s11814-020-0721-0

[CR17] Fiévet F, Ammar-Merah S, Brayner R, Chau F, Giraud M, Mammeri F, Peron J, Piquemal JY, Sicard L, Viau G (2018) The polyol process: a unique method for easy access to metal nanoparticles with tailored sizes, shapes and compositions. Chem Soc Rev 47:5187–5233. 10.1039/C7CS00777A 29901663

[CR18] Fleet ME (1986) The structure of magnetite: symmetry of cubic spinels. J Solid State Chem 62:75–82. 10.1016/0022-4596(86)90218-5

[CR19] Giraldeli LD, Fonseca BC, Reginatto V (2019) Investigating how biomass hydrolysis derivatives inhibit H2 production by an isolated *Clostridium beijerinckii*. Int J Hydrogen Energy 44:14683–14693. 10.1016/j.ijhydene.2019.04.136

[CR20] Hallenbeck PC (2009) Fermentative hydrogen production: principles, progress, and prognosis. Int J Hydrogen Energy 34:7379–7389. 10.1016/j.ijhydene.2008.12.080

[CR21] Heidarian J, Hassan A (2021) Characterization and comparison of fluoroelastomer unfilled, filled with carbon nanotube and carbon black using TGA-GCMS. J Elastomers Plast 53:861–885. 10.1177/0095244321990402

[CR22] Jiang Y, Lu L, Wang H, Shen R, Ge Z, Hou D, Chen X, Liang P, Huang X, Ren ZJ (2018) Electrochemical control of redox potential arrests methanogenesis and regulates products in mixed culture electro-fermentation. ACS Sustain Chem Eng 6:8650–8658. 10.1021/acssuschemeng.8b00948

[CR23] Kumar P, Chandrasekhar K, Kumari A, Sathiyamoorthi E, Kim BS (2018) Electro-fermentation in aid of bioenergy and biopolymers. Energies 11:343. 10.3390/en11020343

[CR24] Lacasse MJ, Zamble DB (2016) [NiFe]-Hydrogenase maturation. Biochemistry 55:1689–1701. 10.1021/acs.biochem.5b01328 26919691

[CR25] Li J, Zhang Y, Sun K, Liu W, Yan H, Meng J (2022) Optimization of a cathodic electro-fermentation process for enhancing co-production of butanol and hydrogen. Energy Convers Manag 251. 10.1016/j.enconman.2021.114987

[CR26] Lin R, Cheng J, Ding L, Song W, Liu M, Zhou J, Cen K (2016) Enhanced dark hydrogen fermentation by addition of ferric oxide nanoparticles using *Enterobacter aerogenes*. Bioresour Technol 207:213–219. 10.1016/j.biortech.2016.02.009 26890796

[CR27] Liu M, Wang Z, Mei J, Xu J, Xu L, Han H, Ni H, Wang S (2016) A facile functionalized routine for the synthesis of imidazolium-based anion-exchange membrane. J Memb Sci 505:138–147. 10.1016/j.memsci.2016.01.036

[CR28] Logan BE, Rossi R, Ragab A, Saikaly PE (2019) Electroactive microorganisms in bioelectrochemical systems. Nat Rev Microbiol 17:307–319. 10.1038/s41579-019-0173-x 30846876

[CR29] Losic D, Farivar F, Yap PL, Karami A (2021) Accounting carbonaceous counterfeits in graphene materials using thermogravimetric analysis. Anal Chem 93:11859–11867. 10.1021/acs.analchem.1c02662 34319694

[CR30] Malode SJ, Pandiaraj S, Alodhayb A, Shetti NP (2024) Carbon nanomaterials for biomedical applications: progress and outlook. ACS Appl Bio Mater 7:752–777. 10.1021/acsabm.3c00983 38271214

[CR31] Meinecke B, Bertram J, Gottschalk G (1989) Purification and characterization of the pyruvate-ferredoxin oxidoreductase from *Clostridium acetobutylicum*. Arch Microbiol 152:244–250. 10.1007/BF00409658 2774799

[CR32] Mishra P, Pradhan N (2024) A synergistic association between iron reduction and enhanced hydrogen production in *Clostridium pasteurianum*. Biochem Eng J 203:109216. 10.1016/j.bej.2024.109216

[CR33] Mostafa A, Im S, Kim J, Lim KH, Kim I, Kim DH (2022) Electron bifurcation reactions in dark fermentation: an overview. Bioresour Technol 344. 10.1016/j.biortech.2021.126327 34785332

[CR34] Mostafazadeh AK, Drogui P, Brar SK, Tyagi RD, Le Bihan Y, Buelna G, Rasolomanana SD (2016) Enhancement of biobutanol production by electromicrobial glucose conversion. Energy Convers Manag 130:165–175. 10.1016/j.enconman.2016.10.050

[CR35] Moura AGL, Rabelo CABS, Okino CH, Maintinguer SI, Silva EL, Varesche MBA (2020) Enhancement of *Clostridium butyricum* hydrogen production by iron and nickel nanoparticles: effects on hydA expression. Int J Hydrogen Energy 45:28447–28461. 10.1016/j.ijhydene.2020.07.127

[CR36] Mulder DW, Shepard EM, Meuser JE, Joshi N, King PW, Posewitz MC, Broderick JB, Peters JW (2011) Insights into [FeFe]-hydrogenase structure, mechanism, and maturation. Structure 19:1038–1052. 10.1016/j.str.2011.06.008 21827941

[CR37] Narcizo JP, Guazzaroni ME, Andrade AR, Reginatto V (2025) Unlocking 1,3-propanediol production by *Pseudomonas aeruginosa* through electro-fermentation. ACS Omega 10(39):45438–45449. 10.1021/acsomega.5c05374 PMC12509004 41078824

[CR38] Patel SKS, Lee JK, Kalia VC (2018) Nanoparticles in biological hydrogen production: an overview. Indian J Microbiol 58:8–18. 10.1007/s12088-017-0678-9 PMC5801174 29434392

[CR39] Qing B, Noori MT, Min B (2025) High-rate biohydrogen production in microbial electrolysis cell using iron-sulfide modified biocathode. Environ Sci Pollut Res 32:5747–5759. 10.1007/s11356-025-35961-z 39951194

[CR40] Selembo PA, Perez JM, Lloyd WA, Logan BE (2009) High hydrogen production from glycerol or glucose by electrohydrogenesis using microbial electrolysis cells. Int J Hydrogen Energy 34:5373–5381. 10.1016/j.ijhydene.2009.05.002

[CR41] Sun Y, Lee J, Kwon NH, Lim J, Jin X, Gogotsi Y, Hwang SJ (2024) Enhancing hydrogen evolution reaction activity of palladium catalyst by immobilization on MXene nanosheets. ACS Nano 18:6243–6255. 10.1021/acsnano.3c09640 38345597

[CR42] Trchounian K, Sawers RG, Trchounian A (2017) Improving biohydrogen productivity by microbial dark- and photo-fermentations. Renew Sustain Energy Rev 80:1201–1216. 10.1016/j.rser.2017.05.149

[CR43] Virdis B, Hoelzle RD, Marchetti A, Boto ST, Rosenbaum MA, Blasco-Gómez R, Puig S, Freguia S, Villano M (2022) Electro-fermentation: sustainable bioproductions steered by electricity. Biotechnol Adv. 10.1016/j.biotechadv.2022.107950 35364226

[CR44] Wang J, Yin Y (2021) *Clostridium* species for fermentative hydrogen production: an overview. Int J Hydrogen Energy 46:34599–34625. 10.1016/j.ijhydene.2021.08.052

[CR45] Yang G, Wang J (2018) Improving mechanisms of biohydrogen production from grass using zero-valent iron nanoparticles. Bioresour Technol 266:413–420. 10.1016/j.biortech.2018.07.004 29982065

[CR46] Zhang Y, Xiao L, Wang S, Liu F (2019) Stimulation of ferrihydrite nanorods on fermentative hydrogen production. Bioresour Technol 283:308–315. 10.1016/j.biortech.2019.03.088 30921584

