# Supplementary material for: Effects of iron nanoparticles and electro-stimulation on biohydrogen production by Clostridium pasteurianum
Source: Appl Microbiol Biotechnol. 2026 Jul 15;110:302. doi: 10.1007/s00253-026-13951-6 (PMC13650106; doi:10.1007/s00253-026-13951-6)
Supplement: Supplementary file 1 [file 253_2026_13951_MOESM1_ESM.docx]

Synergistic and Antagonistic Effects of Iron Nanoparticles and Electro-Stimulation

on Biohydrogen Production by *Clostridium pasteurianum*

Danielli Azevedo Monico*^a^*, Guilherme Bueno Bresciani*^a^*, Adalgisa Rodrigues de Andrade*^a^*, Valeria Reginatto*^a^*^*^.

*^a^Department of Chemistry,* *Faculty of Philosophy, Sciences and Letters of Ribeirão Preto, University of São Paulo, Avenida Bandeirantes, 3900, Ribeirão Preto - 14040-900, São Paulo State, Brazil.*

*Correspondent author: valeriars@ffclrp.usp.br

**Supplementary Material**

**Figure S1**: Gas chromatography of headspace gases from glucose fermentation by *C. pasteurianum*: traditional fermentation without detectable H₂ volume, and traditional and electro-stimulated fermentations with detectable H₂ volume after 6 h.

**Figure S2**: Preliminary evaluation of Fe/CB toxicity under different concentration conditions during a 10 h incubation period.
